# Parkinson Disease-Mediated Gastrointestinal Disorders and Rational for Combinatorial Therapies

**DOI:** 10.3390/medsci4010001

**Published:** 2016-01-20

**Authors:** Syed A. Ali, Ning Yin, Arkam Rehman, Verline Justilien

**Affiliations:** 1 Department of Cancer Biology, Mayo Clinic Cancer Center, Jacksonville, FL 32224, USA; Yin.Ning@mayo.edu (N.Y.); Justilien.Verline@mayo.edu (V.J.); 2 Department of Pain Medicine, Baptist Medical Center, Jacksonville, FL 32258, USA; Arkamr@yahoo.com

**Keywords:** Parkinson disease, neurodegeneration, bowel movements, stem cell, drug combination, targeted therapy

## Abstract

A gradual loss of dopamine-producing nerve cells gives rise to a common neurodegenerative Parkinson’s disease (PD). This disease causes a neurotransmitter imbalance in the brain and initiates a cascade of complications in the rest of the body that appears as distressing symptoms which include gait problems, tremor, gastrointestinal (GI) disorders and cognitive decline. To aid dopamine deficiency, treatment in PD patients includes oral medications, in addition to other methods such as deep brain stimulation and surgical lesioning. Scientists are extensively studying molecular and signaling mechanisms, particularly those involving phenotypic transcription factors and their co-regulatory proteins that are associated with neuronal stem cell (SC) fate determination, maintenance and disease state, and their role in the pathogenesis of PD. Advancement in scientific research and “personalized medicine” to augment current therapeutic intervention and minimize the side effects of chemotherapy may lead to the development of more effective therapeutic strategies in the near future. This review focuses on PD and associated GI complications and summarizes the current therapeutic modalities that include stem cell studies and combinatorial drug treatment.

## 1. Introduction

Parkinson’s disease (PD) is the most common neurodegenerative disorder after Alzheimer’s disease, affecting approximately over six million people worldwide. The key risk factor involved in PD is age and normally a wide range of symptoms appears with ageing [1,2,3]. At first glance, PD appears to be a localized disease, affecting the muscles involved in regulating body motion and characterized by tremor, muscle stiffness and loss of active movements. Often symptoms related to muscular disorders initiate on one side of the body and then during later stages affects both sides of the body [4,5,6]. However, a close inspection of PD reveals that it is not only restricted to impairment of movements but it also has several “offshoot” clinical features.

PD displays a broad spectrum of symptoms and a discussion of all PD related disorders would be beyond the scope of this review article. However, some of the more common symptoms observed in people suffering with PD include (1) *Cognition issues*, related to impaired judgment and loss of memory particularly during latter age [7]; (2) *Sleep disorders* manifested by changes in normal sleeping schedule and time [8]; (3) *Psychiatric problems such as* depression anxiety and stress which add to the motor symptoms. Importantly, stress may cause imbalance of neurotransmitters that can worsen muscular movements related to other activities such as writing, speaking, blinking *etc.* [9]. Furthermore, PD causes issues with the (4) *Autonomous System*, that acts to control the body’s involuntary muscle movements such as heart beating, pupillary dilation, urination, digestion and respiratory tract functions; symptoms including postural hypotension, slowness or absence of movement due to muscle stiffness and constipation may be present during later stage of disease [4,10,11].

Another debilitating symptom of PD is (5) *GI dysfunction*. PD related GI abnormalities appear early in the disease process and significantly add to PD related complications [11,12]. Several GI dysfunctions such as decrease in body weight, deterioration of teeth, gastroparesis and esophageal dysmotility, decrease in bowel movement and defecation are linked to PD [13,14,15]. There is a paucity of clinical data but in general the intensity of these GI symptoms varies from the early to the advanced stage of the PD [16,17,18].

### PD Associated GI Dysfunctions

*Body Weight Loss:* A gradual or sudden loss of body weight appears in 52% of individuals suffering with PD at the early stage of the disease [19]. Weight loss is modest in most of the cases, but can be excessive in severe conditions [19]. The patho-physiological mechanisms of weight loss have not been extensively investigated, however, there are several possibilities that might contribute to the significant loss of body weight in PD pateints. For example, a decrease in energy intake potentially due to olfactory impairment that leads to loss in the sense of taste. For more details readers are directed to descriptive reviews on the topic [20,21,22].

Saliva and sialorrhea: A study in older PD patients determined that saliva pouring while the patient is awake or sleeping occurs approximately in 28% and 58% of the patients respectively. However, it has been proposed that excessive saliva may occur in 70%–78% of patients [23,24]. The increased saliva can affect the muscles regulating esophageal motility through the dorsal motor nucleus of the vagus nerve and sialorrhea can cause aspiration. However, dry mouth due to reduced saliva secretion in the early stage of disease is also common [25,26]. For more details on saliva related symptoms, readers are referred to detailed research articles [26,27,28,29]. Several attempts have been made to develop clinical methods for sialorrhea assessment but there are not well-established accurate and quantitative assays for diagnostic applications. In general, scales are used to evaluate the severity in PD patients [30]. For patients exhibiting severe symptoms, oral drugs such as benzatropin mesylate and trihexyphenidy hydrochloride or periodical injection of botulinum toxin A or botulinum toxin B is recommended. In contrast, for cases with mild symptoms, chewing gum or candy is encouraged [14,31,32,33,34].

*Dysphagia:* Patients with PD may suffer from difficulty or discomfort in swallowing. Dysphagia mainly targets the swallowing tract beginning from preoral, oral, lingual, and pharyngeal to the lower esophageal sphincter [35,36]. Dysphagia involves malfunctioning of the brainstem that mostly results from compromised coordination of oropharyngeal and esophageal muscles. In addition, esophageal dysphagia may also be associated with either Lewy bodies or degeneration of the neurons within the esophageal plexus [37]. Dysphagia occurs in 10%–80% of PD patients and is assessed using barium swallowing tests or video fluoroscopy [38,39]. In the majority of cases dysphagia is detectable in later stages of PD but sometimes it may be present in early stage cases [38,40,41]. In addition to the patho-physiological complications of PD, dysphagia can also result from side effects of PD medications. The typical abnormality associated with PD is the prolongation of swallowing time (double or triple swallow pattern) or a deregulated feeling that could cause abnormal peristalsis and incomplete bolus transit and reduced pressure of the lower esophageal sphincter [42,43,44]. Together, these abnormalities increase the risk of aspiration, and slowed esophageal transit [14]. There is currently no specific treatment approved universally for dysphasia, although in some cases dopaminergic medication can be beneficial [45,46,47].

*Gastric dysfunction:* PD patients not on a treatment regimen develop symptoms related to nausea due to gastric dysfunction *i.e.*, gastroparesis [48]. In a clinical study, untreated PD patients demonstrated an average time to empty half of gastric contents (the half-gastric emptying time) of 59 min when compared to healthy control individuals showing half-gastric emptying time of 44 min [49]. PD patients on dopaminergic medication may also experience nausea. Mild gastric disorder associated with PD can be controlled by consuming small frequent meals, walking 1–2 h after meals and avoiding high fat or fiber-based foods. Also, lying down after meals is recommended if patient has other complications related to blood pressure and heart disease. Pharmacological treatments include domperidone and a peripheral-acting D2 receptor antagonist or intravenous delivery of erythromycin is another approach for the short-term treatment of gastroparesis, but there are concerns about the efficacy of erythromycin and microbial resistance for long-term use [50,51,52].

*Growth of microbes in the small intestine:* A high bacterial density (>105 colony-forming units/mL) in the small intestinal region is associated with malabsorption of food nutrients [53]. Recently, aberrant microbial growth has been implicated as a GI dysfunction in PD, and the prevalence in PD patients ranges from 54% to 67% [54,55,56,57]. Small intestinal floral complication in PD could also be associated with worse motor scores and severe motor fluctuations when on-medication [58]. The treatment of intestinal microbial flora using rifaximin resulted in improved outcomes in PD patients [59].

*Constipation*: Over 50% of PD patients are estimated to display symptoms of mild to severe constipation [60]. The proposed mechanism is slow transit through the colon with, average time found to be twice as long in PD patients when compared to normal controls [60,61,62]. The Lewy body accumulation in enteric neurons may explain changes which may result from altered reflex of the distal smooth muscle due to loss of inhibitory motor neurons. Additionally, anorectal abnormalities because of dopaminergic medications have been reported, demonstrating that this symptom can also be associated with medication [63,64]. Constipation appears at early stage and may persist for several years. A clinical study involving over 6000 male patients without PD showed that risk of PD in later ages increased four times in males that observed less than one bowel movement a day compared to males experiencing more than one movement a day [65]. Interestingly, an autopsy report on PD patients with no clinical data of PD diagnosis suggested that late-life constipation is coupled to incidental Lewy bodies and locus ceruleus as well as reduced substantia nigra neuron density [66,67]. Constipation may be treated with non-pharmacological methods including increased electrolytes, fluid intake, dietary fiber and exercise. In severe cases, pharmacological treatment involves laxatives such as milk of magnesia or polyethylene glycol [68,69].

*Defecation dysfunction*: Clinical symptoms in PD patients appear in more than 60% of cases include colonic inertia to the dysfunction of anal sphincter [70,71]. Excessive pain, incomplete evacuation and stain are the consequence of lack in muscle coordination associated with defecation [41]. Relaxation of various muscles facilitates the expansion of the anorectal angle and fecal expulsion. The problem in defecation occurs when the puborectalis muscle and anal sphincters lose coordination and do not dilate sufficiently enough thus resulting in outlet obstruction [71,72]. This “muscular dystrophy” that leads to the functional obstruction may cause GI pain and a sense of incomplete emptying. The obstruction may also make defecation painful. Therapeutic aspects of defecation dysfunction involve subcutaneous injection of the Botulinum toxin or dopamine agonist into the anal sphincter and pborectalis muscle [60,73,74].

## 2. Prognostic Implications

A progressive loss of nerve cells (neurodegeneration) in the brain is the major cause of PD. The exact cause of “neuronal degradation” is not known. However, the symptoms of disease are associated withthe level of the chemical dopamine, a neurotransmitter produced by nerve cells. Dopamine regulates a variety of functions in the brain that are related to behavior, voluntary movements, punishment, sleep, mood, memory and reasoning. In the case of PD, the dopamine-producing nerve cells gradually die, resulting in decreased dopamine levels and aberrant communication of both motor and non-motor movements [11]. There are several combinatorial factors that cooperate to enhance neurodegeneration. The most common acceptable factors are: genetic predisposition, exposure to a range of heavy metals and chemicals, oxidative stress and the natural cellular life cycle that is part of aging. Extensive clinical studies on human samples have implicated that all patients display Lewy pathology within the enteric nervous system (ENS) [75]. Furthermore, lesions in enteric neurons appear during early stage of the disease prior to the appearance of substantia nigra *i.e.*, neurodegradation. Thus the ENS could be involved in the pathophysiology of the PD disease [48,76]. Dopaminergic nerve cells are abundant in the upper GI tract, whereas their abundance decreases in the large intestine [77]. Although there is a gradient of Alpha Synuclein (α-SYN) neuropathology in the ENS in the early stages of disease with a higher burden in the upper gut when compared to the lower, constipation and defecation dysfunction are the prominent GI symptoms of PD [16]. Examination of the GI tract by using endoscopy is a common way to evaluate accumulation of α-SYN; however, the quantification of α-SYN deposits and determination of a positive result remains ambiguous due to the involvement of various pathology-related technical issues *e.g.*, the population size, site of biopsy, tissue processing techniques, micro-sectioning of the sample and number of subjects [78,79,80].

Treatments related to PD only address the symptoms but not the underlying cause of neurodegeneration. Dopaminergic based drug therapies must be carefully monitored to optimize the reduction in motor symptoms and avoid neuropsychiatric complications. *Deep Brain Stimulation (DBS)* uses a surgical implantation of a highly sophisticated battery-operated device to trigger electrical stimulation to the localized areas of the brain that are responsible for the movement and prevent the unwanted neural signals that regulate tremor. A controlled destruction of the brain’s damaged tissue is still performed in specific circumstances where patients exhibit unilateral tremor [5]. Because the potential of irreplaceable tissue damage is very high, this surgery is used as the last resort. Due to lack of a high number of clinical data and success rates, several therapies are not available for treatments including modern day neuronal SC treatments [81]. Therefore, neuronal research needs to be continued in order to improve our knowledge and understanding of the PD disease that will ultimately lead to the development of more effective treatments.

Treatment of constipation in PD involves increase in both fluid and fiber intake followed by stool softener as a next step if the symptoms have persisted for a long time. If the problem still persists, the doctors do suggest colon-cleansing option such as using MiraLAX^®^, or there have been recommendations of apomorphine injects prior to bowel movement. However, these measures are normally adopted to hasten colon transit and have nothing to do with addressing the PD dependent bowel problem. As a bottom line, GI and urinary tract problems are common features coupled with PD. Awareness of existence and recognition are key steps to successfully address PD associated GI dysfunction. Therefore, patients should not hesitate to share GI-related symptoms with physicians, especially since the knowledge and research of effective treatments is improving.

## 3. Stem Cell-Mediated Therapy in PD

Studies demonstrate an inverse relation between cancer development and PD. However, increasing evidence show a positive association supported by several common genetic mutations that result in cellular changes such as aberrant protein aggregation and cell cycle dysregulation. For details readers are referred to Feng *et al.* [82], but here we briefly discuss an interesting and novel aspect that is related to SC research in PD.

In general, a SC is an undifferentiated cell capable of differentiating into multiple specific lineages. Since SCs have the potential to divide asymmetrically or symmetrically, they, therefore, have potential to replace or restore lost cells when needed. This capacity has been considered as a potential therapeutic approach in disease treatment. SC-mediated therapeutic studies have focused on spinal cord injury, brain ischemia, spinal muscular atrophy, amyotrophic lateral sclerosis and various neurodegenerative diseases [25,83,84,85,86]. We will highlight major advancement in the development of SC-dependent therapy for PD.

Four major types of SC are under consideration for therapeutic purposes, *Embryonic stem cells* (*ESC*) are derived from the inner cell mass of blastocysts and can differentiate into three germ layers and therefore, transplant of ESC has been widely suggested in various neurodegenerative related injuries [87]. In 2006, Kazutoshi Takahashi and Shinya Yamanaka transformed fibroblasts into *induced pluripotent stem cells* (iPSCs), by introducing four specific transcription factors *i.e.*, Oct4, Sox2, Klf4, and c-Myc. Since iPSCs offer less risk of immune rejection and ethical related issues they have recently been used as a potential source to repair neuronal tissues [29,88]. However, one major drawback of the iPSC technology is that the oncogene c-Myc might increase the risk of tumor formation [89]. *Neural stem cells* (*NSCs*) are stem-like progenitors isolated from either fetal brains or specific regions in adult brains [90,91]. NSCs are multipotent SCs that could be differentiated into astrocytes, oligodendrocytes and neurons [92]. Due to lineage restriction, the risk of tumor is reduced, however, NSCs based transplantation is limited because it is challenging to maintain and expand these cells in large numbers [93]. *Mesenchymal stem cells* (*MSCs*) are multipotent cells retrieved from the adult bone marrow [94]. MSCs may also be derived from a variety of non-marrow tissues as well [92,95]. A key property of MSCs is to escape the host’s immune system [73,74]. This characteristic is an important concern for transplantation purposes. Together the current PD therapeutic choices include dopamine agonists, deep brain stimulation (DBS), levodopa and monoamine oxidase inhibitors. However, the therapeutic benefits of oral medications starts to wear-off after five year [96]. Moreover, medication cannot repair the damaged neurons. Therefore, cell replacement therapies may be a valuable therapeutic approach. To achieve a successful SC-based therapy in PD, certain criteria for cell transplantation and the progress of SC-mediated therapies are needed, as discussed in detail in [82,97,98,99].

## 4. Combination Therapies: A Rational for Neuroprotection

Currently there are not many alternatives for the treatment of neurodegenerative. The available therapeutic choices for diseases like PD, PD dementia with Lewy bodies and multiple system atrophy focus on managing the disease symptoms rather than providing a cure. There are positive reports regarding neuroprotection and neuronal SC-mediated treatment, for in depth studies we suggest the following well described research articles [100,101,102,103,104]. However, using strategic drug combinations (multi-target drugs) together with an engineered SC approach might increase the efficiency when compared to mono-therapies. The current advancement in therapeutics support this approach, suggesting that such rational combinatorial therapy may hold promise as the next logical step for PD patient treatment [73,74].

Efforts have been devoted to develop therapies that could either delay or stop PD progression but substantial effective treatments have not been identified yet. This lack of success indicates that conventional approaches are not appropriate for neurodegenerative diseases. Mono-therapies have been effectively used for numerous diseases but might not be significantly effective in patients suffering with neurodegenerative PD. The complex nature of this disorder may require a combination of multi-target drugs that could stop the progression of the disease and improve the patient’s clinical outcome by targeting different signaling pathways that complement each other. The combination of therapeutic approaches has been successfully explored for autoimmune diseases and cancer. However, the combination of pharmacological therapies has not been explored as much as mono-therapeutic efficacies in PD. A more frequent method is the mix of pharmacological treatments with non-pharmacological approaches. Additional basic research is needed to elucidate the molecular mechanism(s) for how drugs interact and how we could exploit their synergy to develop novel therapeutic startegies. Together, preliminary studies suggest that similar combinations would yield better efficacy than using mono-therapies.

There is still no widely used cure for PD since the precise mechanism(s) that lead to the development of PD are not yet well understood. There are high expectations associated with SC-based therapy since many of the cell-dependent *in vivo* studies have shown encouraging results. However, the outcomes in clinical trials have not been consistent because the high self-renewing capacity and pluripotency of SCs lead to risk of tumor formation. Available therapies are mainly focused on managing disease symptom; therefore, the development of new therapeutic strategies for neurodegenerative disorders is urgently needed. This not only includes the development of reliable prognostic Next Generation Sequencing (NGS)-based biomarkers but also of novel therapeutic alternatives that could significantly reduce the progression of PD and PD-related complexities [80]. Recent clinical trials have yielded suboptimal results in favor of individualized medicine, suggesting that combinatorial therapy may be needed to achieve significant improvements. Taking advantage of the potential additive or synergistic effects could be the next logical step for the treatment of PD.
